# A rare case of primary cardiac diffuse large B-cell lymphoma imaged with ^18^F-FDG PET/CT: a case report and literature review

**DOI:** 10.3389/fmed.2024.1373773

**Published:** 2024-03-21

**Authors:** Wenpeng Huang, Zuohuan Zheng, Yongbai Zhang, Yongkang Qiu, Yushuo Peng, Qi Yang, Wei Wang, Lei Kang

**Affiliations:** ^1^Department of Nuclear Medicine, Peking University First Hospital, Beijing, China; ^2^Department of Traditional Chinese Medicine, The Seventh People's Hospital of Chongqing, Chongqing, China; ^3^Department of Pathology, Peking University First Hospital, Beijing, China

**Keywords:** malignant lymphoma, diffuse large B-cell lymphoma, primary cardiac lymphoma, computed tomography, ^18^F-FDG PET/CT, case report

## Abstract

**Background:**

One of the exceptionally rare forms of non-Hodgkin’s lymphoma (NHL) is primary cardiac lymphoma (PCL). The principal clinical manifestation in patients with PCL involves cardiac symptoms resulting from myocardial infiltration by lymphoma, including arrhythmias, heart failure, and chest pain. ^18^F-FDG PET/CT serves as a reliable and indispensable imaging modality for assessing clinically staging NHL.

**Case report:**

We present a rare case involving a 72-year-old woman diagnosed with primary intracardiac diffuse large B-cell lymphoma. For further staging, the patient underwent ^18^F-FDG PET/CT, revealing multiple nodular soft tissue density lesions in the heart and pericardium exhibiting increased FDG metabolism (SUVmax = 12.1). The supradiaphragmatic and infradiaphragmatic segments of the inferior vena cava exhibited irregular morphology with localized nodular changes and increased FDG metabolism in the surrounding area (SUVmax = 9.7). Additionally, multiple enlarged lymph nodes were identified in the left axilla, mediastinum, and adjacent to the abdominal aorta, displaying heterogeneous FDG uptake with an SUVmax of 9.3, indicating lymphoma involvement. The above imaging findings suggested that the mass was a PCL. Hence, the patient underwent a combination of chemotherapy and immunotherapy using R-CDOP (rituximab, cyclophosphamide, liposomal doxorubicin, vincristine, and prednisone). Following two courses of treatment within a span of 2 months, there was a partial remission observed in the cardiac lymphoma and the enlarged lymph nodes.

**Conclusion:**

The case elucidated in this report contributes to an enhanced understanding of the disease for clinicians, with ^18^F-FDG PET/CT providing comprehensive insights into the extent of cardiac involvement, as well as the engagement of extracardiac organs and pathologic lymph nodes. The ^18^F-FDG PET/CT examination not only visually delineates the lesion’s location and extent but also serves as a cornerstone for clinical tumor staging, offering valuable support for treatment monitoring and subsequent follow-up.

## Introduction

Cardiac and pericardial involvement by malignant lymphoma constitutes approximately 1% of cardiac tumors and 0.5% of extra-nodal non-Hodgkin’s lymphoma (NHL), representing a rare phenotype. Diffuse large B-cell lymphoma (DLBCL) is the most common type, but other cell types are also observed, such as T-cell lymphomas (1, 2). Epidemiological characteristics of primary cardiac lymphoma (PCL), according to a recent systematic review conducted in 2020, revealed a mean age of 62, a male preponderance, and a higher prevalence of cases in the Asian region followed by the European region (48% vs. 27%) (3).

One of the exceptionally rare forms of NHL is PCL. Defined by the WHO in 2015, PCL presents as a substantial lymphoma mass involving the heart and/or pericardium, with or without secondary lesions in other areas of the body. The incidence of PCL is a mere 0.056%, with only 10% identified as malignant, accounting for just 1% (4–6). While the right atrium and right ventricle are the most common sites of involvement, occurrences in the left ventricle have also been reported (4, 7). PCL exhibits a broad age range, primarily affecting the elderly and displaying a higher prevalence in men (8); it is more commonly observed in immunocompromised patients (9). The predominant symptoms are cardiac-related, contingent on the anatomical location within the heart. These include arrhythmias resulting from compressed cardiac conducting systems and manifestations of heart failure due to intra-cardiac blood flow obstruction. In some instances, PCL may even mimic myocardial ischemia or myocardial infarction (10). Petrich et al. (4) report constitutional symptoms, heart failure, and pericardial effusion as the most prevalent presenting symptoms and signs of PCL. Lymphoma-related symptoms, such as fever, night sweats, progressive weight loss, and other general systemic manifestations, are uncommon in PCL patients. Third-degree AV block constitutes an infrequent presentation of PCL. Complications may arise from mass effect, local invasion, or embolization (11).

The heart lacks lymphoid nodes, and PCL is believed to originate from the drainage of the epicardial lymph nodes (12). Although the pathogenesis of PCL remains elusive, it is potentially linked to recurrent infections and immune dysfunction, encompassing conditions like human immunodeficiency virus (HIV) infection, Epstein–Barr virus infection, congenital immunodeficiency, and allogeneic bone marrow and solid organ transplantation (2). Diagnosis hinges solely on histopathology and immunohistochemistry for precise identification, classification, and evaluation of proliferative activity (3, 5). Vogl et al. reported a successful CT-guided puncture of the cardiac tumor, enabling a prompt diagnosis of PCL and initiation of therapy without complications (13). Liquid cytology of cardiac or pleural effusion proves highly valuable. It’s essential to note that pericardial effusion cannot be directly attributed to pericardial involvement due to a lack of specific details regarding effusion detection, as cardiac insufficiency may also contribute (3). While DLBCL is the most prevalent histopathology, documented cases include Burkitt lymphoma, T-cell lymphoma, and plasmablastic lymphoma (14). In our case, cytologic analysis of drained pericardial fluid revealed DLBCL, with immunohistochemical staining confirming positivity for CD5. Given the patient’s comorbidities, including coronary artery disease and diabetes, no open chest biopsy was performed.

PCL is an extremely rare malignancy, constituting an oncologic emergency that proves fatal within a few months unless diagnosed and treated promptly (15). The principal clinical manifestation in patients with PCL involves cardiac symptoms resulting from myocardial infiltration by lymphoma, including arrhythmias, heart failure, and chest pain (4, 16). As of now, there is no specific biomarker for the early diagnosis of PCL. 2-Deoxy-2-[fluorine-18]-fluoro-D-glucose (^18^F-FDG) positron emission tomography combined with computed tomography (PET/CT) serves as a reliable and indispensable imaging modality for assessing clinically staging of DLBCL. Despite the rarity of primary cardiac lymphoma, some reports detailing the findings of ^18^F-FDG PET/CT imaging have been presented. Here, we present a rare case involving a 72-year-old woman diagnosed with primary intracardiac DLBCL, referred for ^18^F-FDG PET/CT imaging for staging. According to the World Health Organization (WHO), PCL can be diagnosed if it meets one of the following criteria: (i) primary lymphoma of the heart or pericardium; (ii) lymphoma with the initial cardiac-related symptoms; and (iii) lymphoma dominated by a cardiac mass (17). However, in the early stages, its clinical manifestations do not significantly differ from ordinary chest and heart diseases, often leading to limited attention being paid to the disease when patients present with symptoms such as chest pain or dyspnea (18).

In our comprehensive literature search on the PubMed database spanning from 2009 to 2023, utilizing keywords related to diffuse large B-cell lymphoma and ^18^F-FDG PET/CT, we identified a total of 20 available case reports. The summarized case reports are presented in Table 1.

**Table 1:** ^18^F-FDG PET/CT manifestations of primary cardiac lymphoma.

Case	References	Gender	Age	Clinical symptoms	Primary sites	Invasion and metastasis	Max diameter/cm	SUVmax	Management	Outcome
1	Venugopala et al. (5)	M	55 y	Fever	Pericardium, left ventricle, and right atrium	Superior mediastinum, and mediastinal, right cardio-phrenic, abdominal, bilateral inguinal, axillary, and cervical lymph nodes	4.7	NA	Chemotherapy	Alive at 2 mo
2	Thiagaraj et al. (7)	F	50 y	Abdominal pain, nausea, vomiting, and fever	Left ventricle	NA	4.5	10.7	Surgery + chemotherapy	Alive at 1 y
3	Alansari et al. (10)	M	81 y	Epigastric pain	Right atrium, right ventricle, and intra-pericardium	Lung and liver	4.5	NA	Chemotherapy	Alive at 6 mo
4	Lee et al. (11)	M	51 y	Syncope	Right atrium and right ventricle	NA	NA	NA	Chemotherapy	Alive at 7 mo
5	Imataki et al. (14)	M	62 y	Dyspnea	Right atrium	NA	8.3	NA	Surgery + chemotherapy	Alive
6	Kaida et al. (19)	F	80 y	Dyspnea	Right atrium	NA	NA	14.5	NA	NA
7	Qiang et al. (20)	M	71 y	Syncope, recurrent chest tightness, dyspnea, palpitations, and sweating	Left atrium and left ventricle	Mediastinum lymph nodes	6.7	28.7	Chemotherapy	Alive at 4 mo
8	Seval Erhamamcı et al. (21)	M	70 y	Dyspnea and weakness	Right atrium and auricula	Sternum	NA	26.6	Surgery + chemotherapy	Alive at 3 mo
9	Tong et al. (22)	F	80 y	Chest pain and breathlessness	Pericardium	Bone, peripheral nerves and mediastinal lymph node	NA	NA	Surgery + chemotherapy	Died at 3 mo
10	Chang et al. (23)	F	77 y	Chest tightness and dyspnea	Left atrium, right atrium, and tricuspid annulus	NA	4.9	NA	Chemotherapy	Alive at 3 mo
11	Higgins et al. (24)	F	72 y	NA	Pericardium	Left and right ventricle and brain	6	NA	Chemotherapy	Died
12	Su et al. (25)	M	43 y	Syncope	Interatrial septum, right atrium, and pericardium	Paratracheal, paraaortic regions, and the highest mediastinum lymph nodes	NA	9.24	Chemotherapy	Died at 3 mo
13	Goldman et al. (26)	F	73 y	Shortness of breath, fatigue, fever, and lower extremity swelling	Pericardium, right atrium, and right ventricle	NA	NA	37	Chemotherapy	Alive at 3 mo
14	Takaya et al. (27)	M	53	Penis mass	Penis	Left atrium and right femur	NA	NA	Chemotherapy + stem cell transplantation	Alive at 2 y
15	Franc et al. (28)	F	67 y	Shortness of breath and fatigue	Pericardium, peri-orbit, and axillary lymph nodes	NA	NA	NA	Radiotherapy + chemotherapy + stem cell transplantation	Alive
16	Yang et al. (29)	M	46 y	Abdominal distension, acid reflux, and active chest tightness	Right ventricle, right atrium, stomach, jejunum, and colon	NA	7.8	26.7	Chemotherapy	Alive at 6 mo
17	Tsugu et al. (30)	F	57 y	Low-grade fever and night sweats	Right atrium and uterus	Mediastinum and para-aorta lymph nodes	2.5	22	Chemotherapy	Alive at 3 y
18	Panareo et al. (31)	M	71 y	Dyspnoea and superior vena cava syndrome	Right testicle	Right atrium	8	9.4	Surgery + chemotherapy	Alive at 3 y
19	Tagami et al. (32)	F	76 y	Swelling of the left upper eyelid	Left eyelid	Forehead, nasal cavity, right ventricle, and right atrium	NA	NA	Chemotherapy	Alive at 6 y
20	Kaderli et al. (33)	M	57 y	Mental confusion, weight loss, dyspnea, dizziness, and presyncope with effort	Right ventricle and right atrium	NA	NA	NA	Chemotherapy	Died at 5 mo

## Case presentation

**Figure 1:**
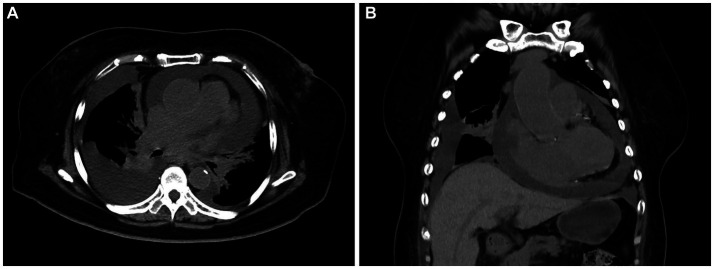
Computed tomography (CT) images of primary cardiac lymphoma (PCL). Transverse **(A)** and coronal **(B)** images revealed multiple nodular and clumped slightly hypodense foci in the heart and pericardium, along with effusions in the pericardium and pleural cavity.

**Figure 2:**
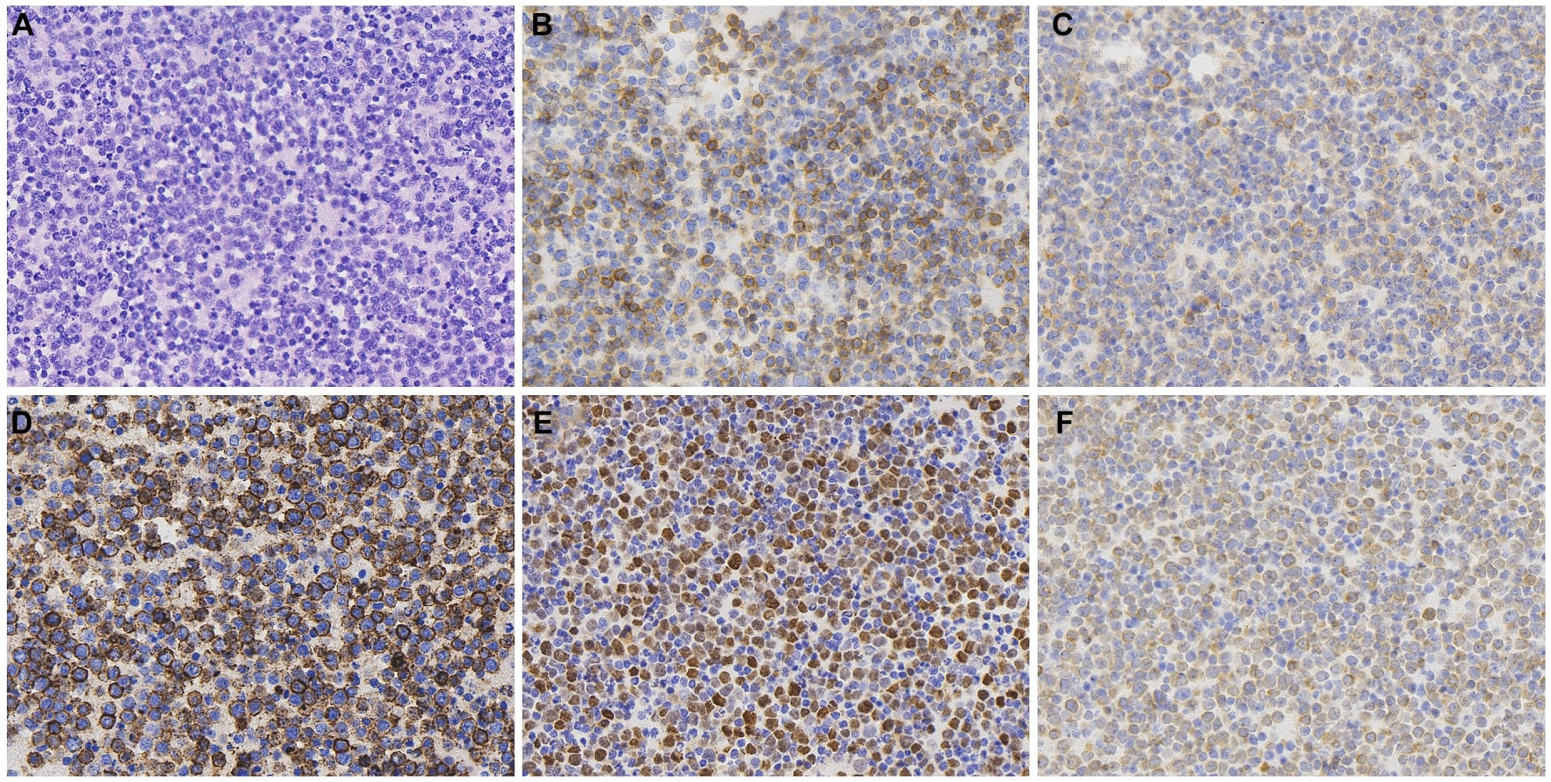
Histopathological and immunohistochemical images of PCL. Hematoxylin–eosin (HE) staining (magnification ×400) of the lesion **(A)** revealed microscopic cells characterized by large, deeply stained nuclei, scanty cytoplasm, moderate cellular anisotropy, visible small nucleoli, and distinct nuclear fission images and apoptosis. Immunohistochemical staining (400×) showed tumor cells were positive for the expression of CD5 **(B)**, CD10 **(C)**, CD20 **(D)**, MUM1 **(E)**, and Bcl-2 **(F)**.

A 72-year-old woman presented with chest tightness and breath-holding symptoms a month before seeking medical attention, aggravated by physical activity and hindering her ability to recline at night. The patient had a history of coronary artery disease and diabetes mellitus; however, she has no family history of genetic disorders or tumors. Acute coronary syndrome was initially ruled out. Laboratory tests revealed CK-MB level at 1.5 ng/mL (normal range: 0–5 ng/mL), high-sensitivity troponin I at 19.3 ng/L (normal range: 0–0.04 ng/mL), and natriuretic peptide at 127.00 pg./mL (normal range: 0–100 pg./mL). Blood gas analysis indicated hypoxemia. The patient initiated diuretic treatment with oral furosemide. However, her breath-holding symptoms intensified after 2 weeks, leading to her hospital admission. An electrophysiology study showed third-degree atrioventricular block. A chest CT scan disclosed an enlarged and dysmorphic heart, displaying multiple nodular and clumped slightly hypodense foci in the heart and pericardium, along with effusions in the pericardium and pleural cavity (Figures 1A,B). The diagnostic imaging physician suspected a malignant tumor of interlobar origin in the heart. Subsequent tumor marker panel analysis revealed elevated CA125 at 203 U/mL (normal range: 0–35 U/mL), CYFRA21-1 at 5.40 ng/mL (normal range: 0–3.3 ng/mL), and NSE at 17.75 ng/mL (normal range: 0–16.3 ng/mL). Cytologic examination of pericardial and pleural effusions exhibited microscopic cells characterized by large, deeply stained nuclei, scanty cytoplasm, moderate cellular anisotropy, visible small nucleoli, and distinct nuclear fission images and apoptosis (Figure 2A). Immunohistochemical staining demonstrated positive expression of CD5, CD10, CD20, MUM1, and Bcl2 (Figures 2B–F). In addition, Ki-67 was observed to be positive in 40% of the tumor cells. *In situ* hybridization remained negative for Epstein–Barr virus (EBV)-encoded small RNAs (EBERs). The pathological diagnosis of cell smears and cell wax blocks in pericardial and pleural effusion confirms DLBCL.

**Figure 3:**
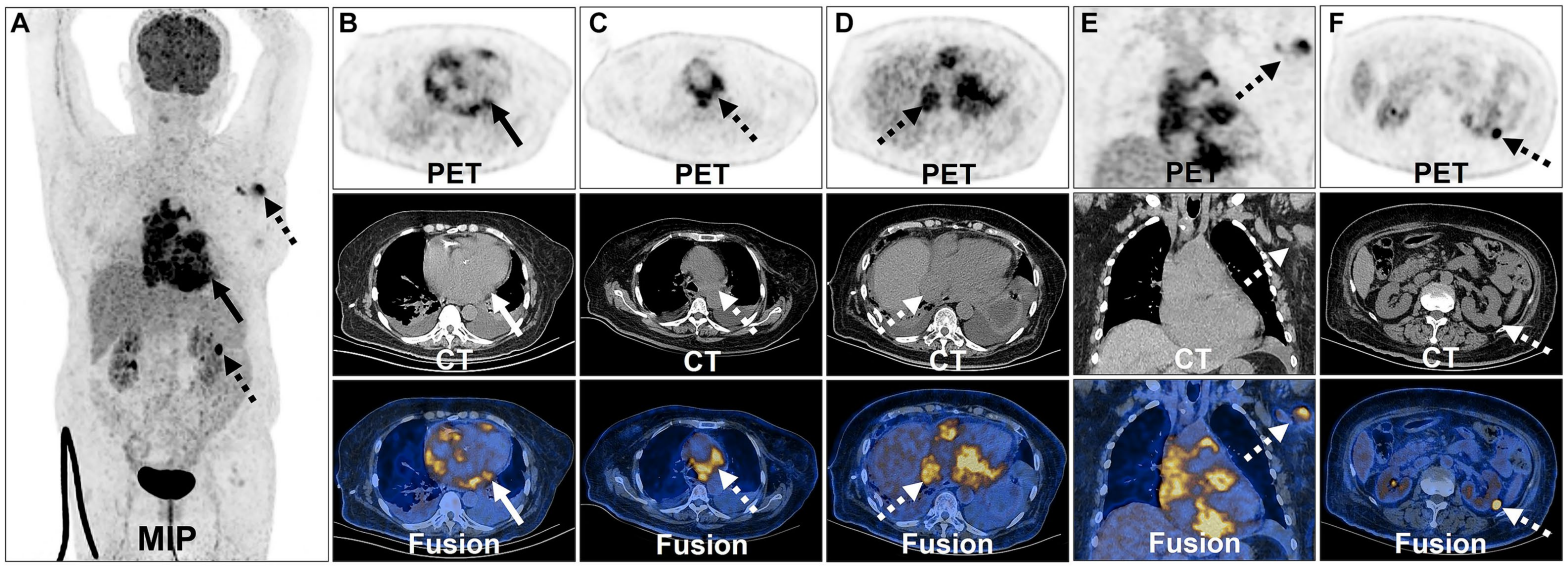
^18^F-FDG PET/CT images of PCL. The anteroposterior 3-dimensional maximum intensity projection image (MIP) demonstrated FDG-avid lesions on the heart, pericardium (long arrows) and multiple lymph nodes (dash arrows) **(A)**. Transverse and coronal images showed multiple nodular soft tissue density lesions in the heart and pericardium exhibiting increased FDG metabolism **(B)**. Enlarged lymph nodes were identified in the mediastinum displaying heterogeneous FDG uptake **(C)**. The supradiaphragmatic segments of the inferior vena cava exhibited irregular morphology with localized nodular changes and increased FDG metabolism in the surrounding area **(D)**. Enlarged lymph node was identified in the left axilla displaying heterogeneous FDG uptake **(E)**. Enlarged lymph node was identified near the left kidney displaying heterogeneous FDG uptake **(F)**.

For further staging, the patient underwent an ^18^F-FDG PET/CT scan. The patient was instructed to follow a high-fat low-carbohydrate diet for 24 h prior to the ^18^F-FDG PET/CT study and to avoid strenuous exercise (34, 35). Additionally, a low carbohydrate meal was advised before starting the 6-h fasting period to maintain blood glucose levels below 11.1 mmol/L. In this examination, 3,000 units of unfractionated heparin was intravenously administered 15 min before FDG administration. The ^18^F-FDG PET/CT scan, performed utilizing Philips GXL-16 PET/CT machines, was conducted 60 min after the intravenous administration of ^18^F-FDG. To ensure optimal hydration, patients were instructed to consume 1,000 mL of water and empty their bladders after 60 min of quiet rest. Routine scans were conducted from the head to mid-thigh, with separate acquisitions of the head and torso. In alignment with the European Association of Nuclear Medicine Research Limited (EARL) standards, we reconstructed the SUVmax for improved reproducibility and comparability (36, 37). This revealed multiple nodular soft tissue density lesions in the heart and pericardium, exhibiting increased FDG metabolism (SUVmax = 12.1). The supradiaphragmatic and infradiaphragmatic segments of the inferior vena cava exhibited irregular morphology with localized nodular changes and increased FDG metabolism in the surrounding area (SUVmax = 9.7). Additionally, multiple enlarged lymph nodes were identified in the mediastinum, left axilla, and near the left kidney, displaying heterogeneous FDG uptake with an SUVmax of 9.3, indicating lymphoma involvement (Figure 3). The above imaging findings suggested that the mass was a PCL.

Hence, the patient underwent a combination of chemotherapy and immunotherapy using R-CDOP (rituximab, cyclophosphamide, liposomal doxorubicin, vincristine, and prednisone). Following two courses of treatment within a span of 2 months, there was a partial remission observed in the cardiac lymphoma and the enlarged lymph nodes. She was closely monitored throughout the chemo-immunotherapy, with an established emergency plan in place to address potential complications, including arrhythmia and cardiac rupture.

## Discussion

Clinical imaging examinations frequently provide crucial insights for the differential diagnosis of a cardiac mass. On CT, PCL typically manifests as multiple iso-attenuating to hypo-attenuating masses infiltrating the myocardium (15, 38). Noteworthy, CT reveals specific anatomical findings of unobstructed coronary arteries. In the case presented in this study, while CT served as the initial diagnostic modality for PCL, it fell short in revealing the details of myocardial and pericardial infiltration. Nevertheless, it did contribute additional information regarding extra-cardiac involvement. Presently, magnetic resonance imaging (MRI) stands as the preferred tomographic modality for investigating PCL, effectively distinguishing the mass from normal myocardial tissue through tissue characterization sequences (39). Beyond its radiation-free nature, MRI boasts advantages over CT, including high contrast and spatial resolution, making it the most useful modality for precisely defining the anatomy of the lesion. Kaida et al. (19) reported PCL to exhibit low signal on T1WI and high signal on both T2WI and fat suppression T2WI. Regrettably, our patient did not undergo a cardiac MRI examination.

^18^F-FDG PET/CT stands out as a noninvasive tool for discerning the metabolic activity of tumors, proving exceptionally beneficial in delineating the staging of malignant lymphoma, tracking relapse, and evaluating therapeutic response (40, 41). When a patient is referred for evaluation of a heart lesion or an area very close to the myocardium, additional dietary recommendations can be helpful. While numerous options exist to reduce normal glucose uptake by the myocardium, common recommendations may include advising the patient to follow a low-carbohydrate diet for 24 h prior to the ^18^F-FDG PET/CT study, or at the very least, consume a low-carbohydrate meal before the 6-h fasting period preceding the study (34, 35). A low-carbohydrate diet helps transition the myocardium from using glucose as its primary energy source to utilizing fatty acids, thereby reducing glucose uptake by the myocardium. Physiological FDG accumulation in the cardiac wall often interferes with the evaluation of cardiac lesions. Ishimaru et al. (42) underscored the significant role of heparin administration before FDG injection in detecting cardiac sarcoidosis. Heparin induces the release of free fatty acids into the circulation and serves to reduce the physiological FDG uptake by the myocardium (43, 44). Plasma glucose levels must be measured before administering FDG. If the plasma glucose level is 11 mmol/L (about 200 mg/dL) or higher, the ^18^F-FDG PET/CT study should be rescheduled or the patient excluded based on individual circumstances and the nature of the study being conducted. For patients with Hodgkin and non-Hodgkin lymphoma, ^18^F-FDG PET/CT has emerged as the standard procedure for staging, monitoring, and restaging the disease. During therapy assessment at mid-treatment and post-completion of chemotherapy, the Deauville score (DS) is recommended for distinguishing between responders and non-responders (45, 46). The DS is a clinical tool used to categorize patients with lymphoma based on the comparison between lesion and reference organ uptake of 18F-FDG, thus reflecting their disease status.

Notably, the existing literature on PCL predominantly comprises case reports, emphasizing its increased metabolic state with substantial FDG uptake (20). PCL may present as either multiple nodular lesions or extensive soft tissue masses (19, 21). The reported SUVmax range for cardiac lymphoma spans from 9.24 to 37 (5, 7, 10, 11, 14, 19–33). Kikuchi et al. (47) underscored a significantly elevated SUV in PCL patients compared to those with other cardiac malignant tumors (e.g., metastases, sarcoma) and benign tumors, demonstrating a lack of overlap. When imaging reveals increased ^18^F-FDG uptake in multiple cardiac tumors alongside significant pericardial effusion, PCL should be a primary consideration. In the case under scrutiny, ^18^F-FDG PET/CT provided comprehensive insights into both the extent of cardiac involvement and the absence of extracardiac organ participation. Another pivotal role of ^18^F-FDG PET/CT in PCL management lies in its ability to monitor treatment response, a critical aspect in restaging numerous common lymphoma types (48). Adequate repeatability and reproducibility are essential for the clinical management of patients and the use of FDG PET/CT within multicenter trials. The new developments in PET/CT have been shown to affect the SUVmax in lesions (49). Consequently, it is conceivable that patients examined on various PET/CT scanners, utilizing different hardware, software, and acquisition parameters, may yield varied SUVmax. In future studies, we recommend that researchers adopt scan acquisition and image reconstruction protocols that are consistent with the EARL Coordination Program (36, 37). This approach will not only enhance the reproducibility and comparability of our findings but also facilitate the meaningful comparison of results across different PET/CT scanners.

In contrast, during the restaging of lymphoma, MRI may persist in exhibiting contrast enhancement, potentially linked to residual inflammation or scar, even when the tumor is no longer metabolically active. Consequently, PET/CT emerged as the preferred modality for assessing treatment response in this scenario. To ensure unequivocal findings, the post-chemotherapy scan was conducted after a 12-h abstinence from a high-fat low-carbohydrate diet, aiming to achieve a favorable tumor-to-background contrast and mitigate potential confounding physiological myocardial uptake in the ^18^F-FDG PET/CT image. Differential diagnoses encompass benign myxoma, the most prevalent type of cardiac tumor, and angiosarcoma, the predominant malignant heart tumor typically located in the left cavities (50–52). While imaging examinations are valuable for detecting and characterizing cardiac masses, arriving at a definitive diagnosis remains challenging. Patient treatment and prognosis hinge significantly on the tissue type and biological behavior of the tumor.

The primary cause of mortality in PCL was heart failure, succeeded by sepsis and the progression of lymphoma. Less frequently observed causes included arrhythmia, embolism, cerebrovascular accidents, and sudden cardiac death (4). Existing strategies for PCL treatment encompass surgery, chemotherapy, radiotherapy, and immunotherapy. Complete resection was deemed unfeasible in our report due to the tumor’s extensive involvement of the pericardium and diffuse infiltration of the myocardium. Early diagnosis and the precise selection of chemotherapy and immunotherapy, guided by cardiac imaging and pathological examination, may significantly improve the prognosis of PCL in atypical locations. Prognostic risk factors include extracardiac involvement, immune deficiency, arrhythmias such as complete atrioventricular block, and left heart involvement (3). The impact of surgical interventions proved limited, necessitating careful consideration of surgical decisions, especially in patients diagnosed with cardiac lymphoma (53). In cases where clinical and radiological suspicions of PCL exist, aggressive diagnostic procedures should be implemented, and therapy initiated before irreversible cardiac damage ensues.

The CHOP (cyclophosphamide, doxorubicin, vincristine, prednisone) regimen remains the conventional choice for treating DLBCL. However, the prognosis of PCL remains unfavorable; Rolla et al. (54) reported on 66 PCL patients, among whom 31 underwent a CHOP-based chemotherapy regimen, resulting in a median survival mean of 7 months. With the widespread use of rituximab (R), the integration of immunotherapy with chemotherapy (R-CHOP regimen) has become the preferred treatment for DLBCL, displacing surgical approaches, regardless of the tumor stage (55). Side effects, observed in approximately 10% of cases, include tumor lysis syndrome and sepsis. Additionally, chemotherapy carries the risk of significant thromboembolism, cardiac wall perforation, ventricular septal rupture, life-threatening arrhythmias, and pericardial effusion. While surgical management does not constitute the primary treatment, prompt surgical debulking is indicated in patients with acute and severe presentations, especially those experiencing rapidly progressing heart failure. Therefore, clinicians must maintain a heightened vigilance index and provide timely interventions to optimize patient prognosis.

## Conclusion

In conclusion, PCL is a rare entity in clinical practice, often associated with a poor prognosis. The advent of ^18^F-FDG PET/CT has marked a significant stride, expanding the potential evaluation capabilities of conventional imaging (MRI and CT). When high ^18^F-FDG uptake is evident in multiple cardiac tumors, accompanied by a considerable pericardial effusion, PCL should be considered a primary diagnostic consideration. The case elucidated in this report contributes to an understanding of the disease for clinicians, with ^18^F-FDG PET/CT providing comprehensive insights into the extent of cardiac involvement, as well as the engagement of extracardiac organs and pathologic lymph nodes. The ^18^F-FDG PET/CT examination not only visually delineates the lesion’s location and extent but also serves as a cornerstone for clinical tumor staging, offering valuable support for treatment monitoring and subsequent follow-up.

## Data availability statement

The original contributions presented in the study are included in the article/supplementary material, further inquiries can be directed to the corresponding author.

## Ethics statement

The patients provided their written informed consent to participate in this study. Written informed consent was obtained from the individual for the publication of any potentially identifiable images or data included in this article.

## Author contributions

WH: Conceptualization, Investigation, Writing – original draft, Writing – review & editing. ZZ: Investigation, Writing – original draft. YZ: Data curation, Writing – original draft. YQ: Conceptualization, Data curation, Writing – original draft. YP: Data curation, Writing – original draft. QY: Conceptualization, Writing – review & editing. WW: Conceptualization, Funding acquisition, Software, Validation, Writing – original draft. LK: Conceptualization, Writing – original draft.
